# Relationship Between Ethical Climate and Burnout: A New Approach Through Work Autonomy

**DOI:** 10.3390/bs15020121

**Published:** 2025-01-24

**Authors:** Carlos Santiago-Torner, Mònica González-Carrasco, Rafael Miranda-Ayala

**Affiliations:** 1 Department of Economics and Business, Faculty of Business and Communication Studies, University of Vic—Central University of Catalonia, 08500 Vic, Spain; 2 Quality of Life Research Institute (Spain), University of Girona (Spain), 17004 Girona, Spain; rafael.miranda@udg.edu

**Keywords:** ethical climate of principles, burnout, work autonomy, emotional exhaustion, depersonalization, ethical climate

## Abstract

Burnout is a factor that affects organizational performance. Researchers have not determined whether an ethical climate and adequate work autonomy provide sufficient emotional stability to dampen burnout or, conversely, increase it. In addition, the abundant literature analyzing the relationship between work autonomy and burnout does not sufficiently establish whether it acts as a stress-reducing resource or a stress-increasing demand. It is also unknown to what extent work autonomy contributes to explaining the relationship between ethical climate and burnout. Therefore, the main aim of this study is to examine the relationship between an ethical climate based on principles and norms and burnout using the moderating effect of work autonomy. We approached this question using a multivariate moderation analysis. The sample consists of 448 employees in the Colombian electric sector. The results show that an ethical climate of principles and work autonomy are positively related to burnout, measured by the dimensions of emotional exhaustion and depersonalization. However, the relationship between an ethical climate of principles and burnout shifts from positive to negative when a rule-demanding work environment is associated with a high perception of work autonomy. In conclusion, when employees have considerable control over their usual tasks, they develop a pattern of behavior that incorporates both the organization’s internal standards and the principles that shape individual morality. In this case, employees are able to balance the workload with the high psychological demands of an ethical climate of principles, without it being a disturbance to their emotional well-being. The results of this research are particularly relevant because they show for the first time that an ethical climate of principles can have opposite effects on employee burnout, both positive and negative, depending on the degree of work autonomy. This opens the door to new strategies for organizations to prevent certain psychosocial occupational risks, such as burnout, which often have a serious impact on employees’ mental health. Moreover, the model of moderation proposed in this article can be replicated in other Latin American countries with similar characteristics to those of Colombia or even transferred to rich countries.

## 1. Introduction

To our knowledge, the relationship between ethical climate and burnout, although it has aroused considerable interest in occupational health sciences, remains unclear. For example, Bao et al. (2013), Barr (2021), Huhtala et al. (2022), Maffoni et al. (2022), Plouffe et al. (2021), and Rivaz et al. (2020) highlighted ethical climate as a protective factor for burnout. In contrast, Dal Corso et al. (2019) and Tehranineshat et al. (2020) suggested that ethical climate is a predictor of burnout. These contradictory results might be explained by the lack of publications that have examined the underlying mechanisms that strengthen or weaken the relationship between highly regulated ethical climates (e.g., principles) and burnout, for which the inclusion of other related variables turns out to be crucial. In this regard, Ghasempour Ganji et al. (2021) considered the role of job autonomy as a necessary condition for employees to be able to interpret the rules within an ethical scenario. What follows is a description of ethical climate, burnout, and work autonomy, together with the state of research on these topics, with a particular focus on the links between them, leading to the identification of the gap that this research aims to fill, namely, the lack of studies on the role of an ethical climate of principles on employees’ emotional well-being.

Ethical climate is defined as a shared understanding of what behaviors are ethically correct and how to deal with behaviors that are ethically deviant. An ethical climate is an environment that fosters ethical thinking, trust, and mutual respect in an organization and allows both the questioning and expression of different opinions (Borrelli et al., 2023). Being associated with the promotion of positive behaviors and the prevention of irregular or deviant work behaviors, it plays a key role in organizational regulation and is indispensable for interpreting the emotions, feelings, and attitudes of individuals (Elçi & Alpkan, 2009; Elçi et al., 2015; Teresi et al., 2019). Therefore, the analysis of the ethical climate of the workplace is fundamental to assessing the influence of ethics on workers’ health (Borrelli et al., 2023).

Several authors have highlighted the benefits associated with ethical climates, for example, Sillero-Sillero et al. (2023) suggested that promoting an ethical climate in the work environment is related to improved employee satisfaction and commitment, which are key factors in increasing happiness and work performance (Asgari et al., 2019; Okpara & Wynn, 2008; Sillero-Sillero et al., 2023). Similarly, Ayub et al. (2022) established a negative relationship between ethical climate and burnout through the theory of role stress. According to this perspective, an ethical climate is a key factor that mitigates the effects of ambiguity in work roles and prevents excessive emotional tension. In the same line, Rivaz et al. (2020) and Saleh et al. (2022) argued that an ethical climate functions as a context that counteracts the negative effects of emotional exhaustion and depersonalization, thanks to a high perception of organizational support. However, the first study analyzed ethical climate in general terms. That is, it does not propose a differentiation between its dimensions. The second study only analyzed ethical climate at an individual level, i.e., ethical reasoning is located within the individual and is alien to the rest of the organization. Therefore, key aspects such as cooperation, group interest, or external laws and codes are not taken into account (Saleh et al., 2022).

In contrast, some studies showed the negative effects associated with ethical climates, for example, Atabay et al. (2015) observed a positive correlation between a rules-based ethical climate and moral discomfort. An ethical climate whose primary concern is norm compliance and specific organizational regulation criteria builds a work environment that prevents employees from feeling autonomous (Lee & Wu, 2024). This rigidity may clash with the employees’ perceptions of autonomy and be associated with higher levels of moral distress (Noh & Kim, 2024). In fact, moral distress arises when personal ethics conflict with institutional constraints, such as autonomy. What employees consider correct faces organizational barriers, and this situation limits the ethical course of action they deem appropriate. Under such circumstances, moral distress often leads to a growing concern among organizations, which is burnout (Dzeng & Curtis, 2018). Burnout is a psychological state that affects the way people perceive themselves and others at work and has been recognized by the World Health Organization as an occupational disease. It is divided into three dimensions: emotional exhaustion, depersonalization, and personal realization (Borrelli et al., 2023). Emotional exhaustion is defined as a sustained decline in the reserves that workers have. This context leads the employee to become emotionally distanced from work and to gradually reduce his or her performance. Depersonalization is defined as the gradual distancing of the employee from the rest of his colleagues, leading to a decrease in his emotional capacity. Finally, personal realization refers to the level of satisfaction employees feel with their job performance (Maslach et al., 2001). The study of burnout in organizations is important because it is an occupational health problem that, in addition to causing negative consequences at work, can generate feelings of helplessness, negative attitudes toward others, low self-confidence, and a generalized loss of interest in the organization (Elçi et al., 2015; Teresi et al., 2019).

The research presented here follows the approach of Elçi et al. (2015), omitting the evaluation of the perception of personal realization as it is considered a consequence of the first two dimensions. According to Elçi et al. (2015), burnout is a significant factor in employee behavior and represents one of the most damaging outcomes at the organizational level. In that sense, the direct relationship between an ethical climate and burnout can be a key indicator of the occupational health of any organization. Following Tomczak and Kulikowski (2024), burnout frequently results in serious consequences for both individuals and the organization. These consequences can include issues affecting the mental or physical health of employees, absenteeism, high turnover, and a strong sense of inability to adequately address a problem that negatively affects workers’ performance. From this perspective, the impact of specific ethical climates, e.g., principled, on burnout is an emerging issue that requires further research due to the lack of theoretical and practical evidence.

Victor and Cullen (1988) suggested that an ethical climate of principles is nurtured not only by personal ethics but also by organizational rules and procedures along with the legal system in place in the country. In other words, an ethical climate of principles must be aligned with the moral personality of employees in order to be efficient. Consequently, when an ethical climate of principles imposes moral obligations that may disregard or conflict with the employees’ values, it triggers a process of moral demotivation, as they perceive this situation as a demand (Besser-Jones, 2008). This disproportionate context can have strong emotional impacts that overwhelm employees and cause them to lose valuable personal resources. The conservation of resources theory (COR) by Hobfoll and Lilly (1993) proposed that when individuals exercise their profession under difficult emotional conditions and depleted coping resources, they commonly enter a spiral of energy loss and mental fatigue.

Autonomy is the individual’s ability to choose how a particular process or task is carried out, and it is for this reason that work autonomy has a positive impact on individuals in a number of ways. First, it emotionally relieves employees and gives them the authority to decide and choose the best way to perform tasks according to their own criteria (Zhang & He, 2022). Work autonomy shows an inverse relationship with emotional exhaustion, which helps prevent burnout by significantly affecting work satisfaction (Farfán et al., 2020). In this sense, the job demands–resources theory suggested by Bakker and Demerouti (2017) considered that work autonomy is a resource that only affects work results positively. However, this positive impact depends not only on employees accepting greater job responsibility but also on their level of self-efficacy. The self-determination theory by Deci et al. (2017), applied to the organizational context, suggested that work autonomy is a primary job characteristic that is related to individual interests and values. In that sense, Ryan et al. (2021) suggested that when employees have a strong perception of autonomy, they find ways to satisfy their basic needs for competence and relatedness. In addition, Liang et al. (2024) considered that work autonomy buffers a scenario of abusive supervision.

However, work autonomy can represent an opportunity or an obstacle for employees’ emotional well-being. Autonomy is, in principle, a key aspect of both occupational health and human motivation (Ryan & Deci, 2019). However, the perception of self-control becomes a psychosocial risk when limits, about the possible demands, between control and resources are not clearly established. When the organizational climate is based solely on norms, which can be perceived as demands, and the work schedule is not clearly defined, work intensity, task overload, and time pressure may become part of the employees’ daily lives (Pérez-Zapata et al., 2016). This context, which is not necessarily voluntary, poses a risk that may ultimately translate into burnout.

### 1.1. Contextualization of the Study

In most developing countries, corruption and political opportunism are important factors that often lead to inefficiency throughout the national territory, especially in the electricity sector. The Colombian electricity sector has wanted to break with these negative dynamics since its current situation demands that organizational rules and norms become the basic principles of its internal restructuring. The Colombian electricity sector is one of the main axes on which the country’s development is based due to its contribution to the generation of employment and by financing an important part of the national budget that is dedicated to social investment. The creation of a collective action on ethics and transparency was born as an initiative that aims to transform the Colombian electricity sector through good practices and a strict regulatory framework that strengthens the ethical principles of employees (Santiago-Torner, 2023a). Consequently, there is a clear need to know if a climate defined by demanding norms and by breaking with previous paradigms has a positive or negative effect on the emotional health of employees. In addition, for the Colombian electricity sector, it is also critical to know whether work autonomy has an impact on this relationship since its workforce, with a high level of university education, is characterized by being granted well-paid jobs and strong autonomy.

### 1.2. Objective and Hypotheses

Taking the above considerations in mind, the objective of this study is to analyze the relationship between an ethical climate of principles and burnout by considering the moderating effect of work autonomy in the Colombian electricity sector. In coherence with this objective, three different hypotheses have been formulated departing from the fact that, first of all, a rigid climate focused on norm compliance is usually subject to high psychological demands and low perception of support (Borry, 2017; McLinton et al., 2023). Besides affecting employee motivation, this mismatch between resources and demands can lead to negative emotional processes that significantly harm employee health. Strict discipline and imposing a climate aimed at rules compliance induce more bureaucratic and impersonal processes and often result in repetitive and stressful work. In fact, an environment with weak feedback is likely insufficient to develop new values that change behaviors internalized by employees (Lindeberg et al., 2011). When an ethical climate of principles proposes moral obligations that possibly ignore or disagree with the employees’ values, a process of moral demotivation opens as this situation is perceived as a demand (Besser-Jones, 2008). This disproportionate context can have strong emotional impacts that overwhelm employees and cause them to lose valuable personal resources.

In that sense, Hobfoll and Lilly (1993) proposed that when individuals practice their profession under difficult emotional conditions and with depleted coping resources, they commonly enter a spiral of energy loss and mental fatigue. Therefore, the following hypothesis is proposed:

**H1.**  *An ethical climate of principles will be positively related to burnout, controlling for gender and seniority.*

Secondly, since work autonomy is a key factor in proper job design (Zhang & He, 2022), the tension between autonomy and control becomes more evident in organizational climates that pressure employees to conform to normative expectations. This intentional constraint can undermine the beneficial approach that many authors attribute to flexibility in performing tasks (Farfán et al., 2020). Physical and emotional strain increases as job demands become more evident and employees lack sufficient resources to cope with them successfully. According to Luna et al. (2023), the degree of autonomy may be a detrimental factor to the emotional well-being of professionals. Work autonomy may be related to certain work conditions, including lack of support and time pressure, as common detrimental effects (Kubicek et al., 2017). Indeed, when a work environment focuses on the pursuit of uniform codes of conduct, it exerts external control over employees. The increasing need to monitor the achievement of specific goals and project the value or direction of work become demands that generate stress, ambiguity, and uncertainty in highly regulated environments (Johlke & Iyer, 2013). In that sense, Mazmanian et al. (2013) specified that a higher level of autonomy implies a greater dedication to work, which leads employees to lose control over their lives in favor of the employing entity (Shevchuk et al., 2019). This situation can have important implications for employees’ work–life balance. Therefore, work autonomy can lead to stress levels that result in burnout. Consequently, the following hypothesis is proposed:

**H2.**  *Work autonomy will be positively related to burnout, controlling for gender and seniority.*

Thirdly, the true nature of an ethical climate of principles considers the moral values of employees and not just organizational norms. This integrative approach prevents the role conflict that arises when personal values are misaligned with organizational expectations. In fact, providing clear signals about institutional ethical intentions becomes a key aspect of reducing stress levels if employees consider them acceptable (Mulki et al., 2008). Emotional energy is depleted not only by demanding working conditions but also by not having sufficient resources to support the work. In this sense, both the conservation of resources theory (Hobfoll et al., 2018) and the job demands–resources theory (Bakker & Demerouti, 2017) consistently suggest that the loss of resources results in permanent stress leading to burnout. However, the importance of an ethical climate to facilitate or hinder the achievement of specific basic needs, such as autonomy, remains difficult to assess. According to the self-determination theory (Adams et al., 2017; Deci et al., 2017; Ryan & Deci, 2000), autonomy is a work resource that activates and guides human behavior by covering a basic psychological need. Therefore, autonomy is an aspect of work that can explain the relationship between job demands and burnout (Fernet et al., 2013). This theory suggests that some external factors, e.g., excessive supervision or a very rigid work climate, tend to decrease the sense of autonomy, causing a shift in perceived locus of control from internal to external (Deci et al., 2017; Ryan et al., 2021).

When an ethical climate of principles is able to integrate the employee’s moral character with organizational rules, taking into account the importance of individual values, it limits uncertainty, strengthens the perception of internal locus of control, and avoids a possible breach of the rules (Borry, 2017). For this reason, a climate with a less rigid orientation that provides the employee with the necessary tools to face an ethical dilemma can protect his or her emotional well-being by satisfying the basic need for autonomy.

In fact, when employees perceive that the environment surrounding them does not limit or pressure their behavior or way of thinking, their psychological resources improve through a greater feeling of autonomy. In this sense, work autonomy can activate different motivational processes and a certain psychological freedom that prevents burnout. On the other hand, rules linked to an ethical climate of principles can prevent employees from being exposed to a succession of overwhelming demands that exceed both their autonomy and emotional limits, leading to burnout (Edú-Valsania et al., 2022). Examples of such overwhelming demands may include role overload, work settings with excessive work, or abusive leadership styles.

Although we expect a positive relationship between an ethical climate of principles and burnout, we suggest that this positive effect will be attenuated when there is an adequate interaction between organizational norms and work autonomy. That is, the employee’s understanding and internalization of the regulatory aspects proposed by the organization will enhance the positive effect of work autonomy. Therefore, the intersection between norms and autonomy will determine when or under what circumstances (moderation) the positive relationship between an ethical climate of principles and burnout diminishes or even stops (Rego et al., 2019). Consequently, the following hypothesis is proposed:

**H3.**  *Work autonomy will inversely moderate the positive relationship between an ethical climate of principles and burnout, controlling for gender and seniority.*

This study aims to contribute to the existing literature on occupational health in different ways. Firstly, there are very few studies that have not been developed among healthcare workers in rich countries. Therefore, the present study carried out with employees in the electricity sector in an emerging country, such as Colombia, constitutes a novelty. Secondly, our results may provide a previously unstudied perspective on the relationship between an ethical climate of principles and burnout by considering also the negative impact the former might have on the latter. Thirdly, our results can make a significant contribution to the field of occupational health and safety since increased work autonomy is not necessarily associated with increased psychological well-being. Finally, and most importantly, this study aims to provide evidence, in a novel way, of the extent to which an ethical climate of principles that integrates moral-related behaviors and rules, can be a motivational force capable of preventing the emergence of emotional exhaustion and depersonalization through different perceptions of work autonomy (Zhang & He, 2022). This opens the door to new strategies for organizations to prevent certain psychosocial occupational risks, such as burnout, which often have a serious impact on employees’ mental health.

## 2. Materials and Methods

### 2.1. Data Collection and Sample

The study is cross-sectional, correlational, non-experimental, and quantitative, based on the use of a questionnaire as the only data collection technique. A cluster sample was used, including Colombia’s main cities (clusters). The sample selection of organizations had a confidence level of 95%. Most of the companies in the Colombian electric sector concentrate in the five capitals of the country’s key departments (Cundinamarca, Antioquia, Valle del Cauca, Risaralda, and Caldas). The research project was presented at the Colombian electric sector’s community action meeting in mid-2021. More than 50% of the 35 selected organizations in the first instance expressed interest in the study, precisely 18. The selection process of the organizations took into account factors such as market share, location, seniority, and the number of employees. Ultimately, their sector visibility and countrywide location led to the selection of six collaborating organizations out of 18. Data collection took place between October and December 2021. We established only two selection criteria when choosing the participants: a minimum of one year of seniority and an indefinite contract type. These criteria aimed to guarantee that the employee understood the work environment and that a fixed-duration contract did not influence their responses.

During this second stage, confidentiality agreements were signed, and the following documents were sent: voluntary consent and waiver, data protection, and objectives presentation. The questionnaire was supervised by a group of experts and sent to the participants online using the Google Forms tool. The survey was written in Spanish by a bilingual researcher using the conventional “back translation” method (Brislin, 1980). All the research was subject to an ethics committee at the end of 2021. The survey took an estimated time of about 35 min to be completed.

We took into account the suggestions of Podsakoff et al.’s last article (Podsakoff et al., 2024) to minimize the effects of common method bias (CMB). Firstly, we collected data from various organizations. This technique decreases biases associated with transient mood states and also reduces the tendency to respond in a socially desirable manner. Second, the principal investigator was present in all six data collection processes. The participating organizations allocated one hour of work time for their employees to complete the survey. To eliminate possible ambiguity in the items on each scale evaluated, the principal investigator provided additional information to make the questions in the questionnaire as simple and specific as possible. Third, the principal investigator guaranteed the anonymity of the respondents. The objective of this technique is to reduce the distrust or fear that the respondent may experience when giving personal information.

The final sample is formed by 448 professionals who work in the Colombian electric sector, specifically in six organizations with central offices in Bogotá, Cali, Medellín, Manizales, and Pereira. Regarding gender, 175 (39%) participants were women, and 273 (61%) were men. The average age is 37.18 years (SD = 10.059; range: 20–69). A total of 448 professionals have permanent work contracts (100%), and mean seniority in the organization is 13.06 years (SD = 8.82; range: 1–38 years). Regarding work activity, 86.6% (308) are analysts, 8.9% (40) have intermediate jobs, and 4.5% (20) are managers. All (100%) of survey participants have university studies, and 57.4% (257) have graduate studies. Approximately 58% (260) have children.

### 2.2. Instruments

Control Variables: Control variables improve the internal validity of a study by limiting the influence of confounding variables. A confounding variable is a variable that is not adequately controlled for in the study and that may affect the true association between the independent variable and the dependent variable. Therefore, the use of adequate control variables helps to establish an optimal correlational relationship between the variables of interest and also avoids biases associated with the research (Newey & Stouli, 2021).

In this research, seniority and gender are considered control variables since they can potentially affect the results. Specifically, seniority may be a critical occupational factor linked to chronic stress (Vargas et al., 2014). Furthermore, it is possible to assume that women experience higher levels of emotional exhaustion than men. The imbalance in domestic workloads between men and women is likely to be a key factor justifying women’s greater physical and mental exhaustion (Artz et al., 2022). To measure seniority, survey participants were asked to indicate how long they had been working using a scale with one year as the minimum. Gender was coded as 0 for men and 1 for women.

Ethical climate based on principles: One of the subscales proposed by Victor and Cullen (1988) was used to evaluate the ethical climate of principles, composed of 11 items organized into three subdimensions: (1) personal morale (three items), (2) rules and procedures (four items), and (3) laws and professional codes (four items). This research used a six-point scale instead ranging from “strongly disagree” to “strongly agree”. A six-point scale is used because an even number of responses produces uniformly categorized results, which facilitates understanding and discussion of the results (Taherdoost, 2019). Items for example include “Individuals are expected to follow the law and professional standards above other considerations”. The present research obtained a Cronbach’s alpha of 0.74. Likewise, composite reliability (CR) and average variance extracted (AVE) were calculated. The results indicate that CR is optimal (CR = 0.74) and AVE is adequate (AVE = 53%). According to Bagozzi et al. (1998) and Chin (1998), these two values are relevant as they are above 0.70 and 50%, respectively.

Work Autonomy: The one-dimensional scale suggested by Spreitzer (1995) was used to measure work autonomy using three items. It assesses whether employees have sufficient independence to decide the direction and intensity of their efforts when performing their work and to assume firm control over it. The present research used a six-point scale instead ranging from “strongly disagree” to “strongly agree”. Items for example include “I can decide on my own how to do my work.”. This research reaches a Cronbach’s alpha of 0.87. Additionally, results indicate that CR is optimal (CR = 0.73), and AVE is adequate (AVE = 80%).

Maslach Burnout Inventory: Multidimensional model that differentiates between emotional exhaustion and depersonalization.

(1). Emotional Exhaustion: Emotional exhaustion was measured using the five items proposed by Schaufeli et al. (1996). The effect of the workload on individuals’ emotional resources is evaluated. This research used a six-point scale ranging from “strongly disagree” to “strongly agree”. Items for example include “I am emotionally exhausted at my job”. This research achieved a Cronbach’s alpha of 0.90. The results indicate that CR is optimal (CR = 0.81), and AVE is adequate (AVE = 68%).

(2). Depersonalization: Depersonalization was measured using the four items proposed by Salanova and Schaufeli (2000). It assesses whether the workload gradually consumes employees’ emotional resources until a distance is created between them and the rest of the organization’s members. This research used a six-point scale ranging from “*strongly disagree*” to “*strongly agree*”. Items for example include “I have become more cynical about whether my work contributes anything”. The present research achieved a Cronbach’s alpha of 0.90. The results indicate that CR is optimal (CR = 0.86), and AVE is adequate (AVE = 66%).

### 2.3. Data Analysis

First, descriptive analyses of the evaluated variables were conducted, including means, standard deviation, kurtosis, and skewness. Second, a correlation analysis between the study variables was performed, including the calculation of discriminant validity. Discriminant validity is a type of construct validity that assesses whether the variables or measures used are independent and unique. Discriminant validity depends on the square root of AVE being greater than the different correlations between variables (Fornell & Larcker, 1981).

Third, confirmatory factor analysis (CFA) is a statistical technique used to verify the factor structure of a set of observed variables. CFA allows testing the hypothesis that there is a relationship between the observed variables and their underlying latent constructs. CFA is usually applied to scales based on theoretical models whose validity and reliability have been previously tested. In fact, the aim is to know the validity of the theoretical model based on these scales for a specific study, without determining causality between the variables (Galindo-Domínguez, 2019).

Fourth, we used the AMOS macro included in the SPSS statistical package to perform the moderation process. SPSS Amos is a software that allows us to create structural equation models (SEMs) (Sürücü et al., 2023). SEM is a multivariate statistical analysis technique that allows us to simultaneously analyze all variables in the model, examine complex patterns of relationships between variables, compare between and within groups, and validate theoretical and empirical models. In addition, the mediation error is not aggregated into a residual error term. Moderation analysis is a type of multivariate analysis in which an independent variable predicts a dependent variable by taking into account the causal strength of a third variable that interacts between the two, called the moderating variable (Galindo-Domínguez, 2019). In this line, the moderating variable affects the strength and/or direction of the relationship between the predictor variable and the output variable. Unlike mediation analysis which tries to quantify how or why a certain phenomenon occurs, moderation analysis tries to quantify especially when or under what circumstances it occurs.

Before the moderation analysis, the data were checked in terms of linearity, normality, and multicollinearity issues. Kurtosis, asymmetry, and Mahalanobis distance scores were examined to determine linearity and normality. Variance inflation factors (VIFs) and Condition Index were used to assess multicollinearity issues. Condition Index values must be below 30 and VIF values below 10 to meet the assumption of normality. Outliers were verified using Mahalanobis distances as suggested by Aguinis et al. (2017). No multicollinearity issues were identified, and the data were normally distributed.

## 3. Results

### 3.1. Preliminary Analyses

#### 3.1.1. Descriptive Statistics

Table 1 shows the descriptive analyses. Negative skewness values indicated a slight rightward skew in the data distribution. Kurtosis values below 2 indicated a distribution similar to the normal distribution. However, the positive sign indicated a leptokurtic distribution. Indeed, data values were closer to the mean and outliers were rarer. Generally, the data are considered normal when kurtosis is between −7 and +7, and skewness is between −2 and +2 (Demir, 2022; Jammalamadaka et al., 2021).

The correlation analysis conducted showed that the ethical climate of principles (ECP) showed a positive relationship with WA, EE, and depersonalization (DE). Finally, WA was related to EE and DE (see Table 2).

#### 3.1.2. Confirmatory Analysis

We performed confirmatory factor analysis (CFA) to give empirical validity to the conceptual structure of this research. To evaluate the goodness of fit, we analyzed that (a) the SBχ2 index over the degrees of freedom (SBχ2/gl) was less than 5; (b) the Goodness of Fit Index (GFI), the Comparative Fit Index (CFI), the Tucker Lewis Index (TLI), the Normalized Fit Index (NFI), and the Incremental Fit Index (IFI) reach values equal to or greater than 0.90; (c) the Root Mean Square Error of Approximation (RMSEA) was less than 0.05 (Hair et al., 2016).

The theoretical model obtained good results. The incremental fit indices provided excellent values (NFI = 0.91; TLI = 0.91; CFI = 0.92; IFI = 0.94). On the other hand, the absolute fit indices showed acceptable values (RMSEA and GFI 0.076 and 0.92, respectively). Finally, AIC, ECVI, and the χ2/gl ratio presented optimal values: 645.19, 1.44, and 3.56, respectively. Although RMSEA values below 0.05 are considered a good fit, values up to 0.08 represent reasonable errors of approximation (Byrne, 2001).

### 3.2. Moderation Analysis

**Figure 1:**
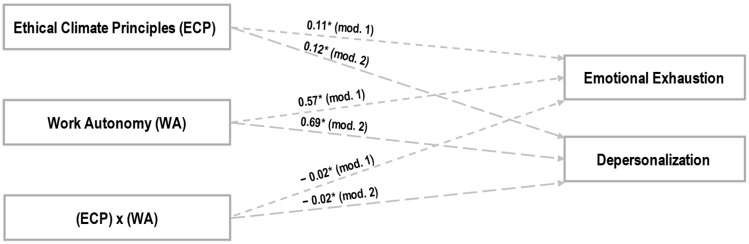
Analysis results, model 1 (mod. 1) and model 2 (mod. 2). * *p* < 0.05.

Figure 1 and Table 3 and Table 4 reveal the results of the double analysis of simple moderation. The coefficients used are not standardized. Bootstrapping samples are over 10,000, and confidence intervals are around 95%. The LLCI and ULCI values operate as lower and upper bounds. *Hypothesis 1 proposed that an ethical climate of principles will be positively related to burnout, controlling for gender and seniority*. The linear regressions corresponding to effect a1i of model 1 (β = 0.11, SE = 0.15, *p* = 0.01) and of model 2 (β = 0.12, SE = 0.17, *p* = 0.01) justify this assumption (see Table 3 and Table 4). *Hypothesis 2 proposed that work autonomy will be positively related to burnout, controlling for gender and seniority*. The linear regressions corresponding to effect a2i of model 1 (β = 0.57, SE = 0.25, *p* = 0.01) and of model 2 (β = 0.69, SE = 0.35, *p* = 0.01) validate this assumption (see Table 3 and Table 4). *Hypothesis 3 proposed that work autonomy will inversely moderate the positive relationship between an ethical climate of principles and burnout, controlling for gender and seniority.* The linear regressions corresponding to effect a3i of model 1 (β = −0.02, SE = 0.05, *p* = 0.01) and of model 2 (β = −0.02, SE = 0.04, *p* = 0.01) confirm this assumption. Low, medium, and high indirect effects confirm work autonomy’s moderating effect (see Table 3 and Table 4).

Figure 1 shows the proposed statistical diagram and the results of the simple moderation.

**Figure 2:**
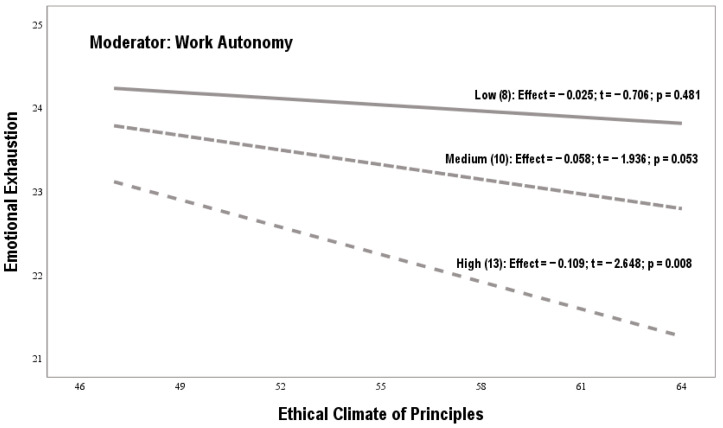
Moderating effect of the work autonomy variable (model 1).

Figure 2 shows the moderating effect of work autonomy on the relationship between an ethical climate of principles and emotional exhaustion. The macro-PROCESS provides three independent values (low, medium, and high). The graph shows that low and medium autonomy levels were not statistically significant. In other words, these two autonomy levels did not influence the impact of an ethical climate of principles (X) on emotional exhaustion (Y). However, a high perception of autonomy changes the meaning of the relationship between X and Y. Therefore when an ethical climate of principles coexists with high autonomy, it has a buffering effect on emotional exhaustion.

**Figure 3:**
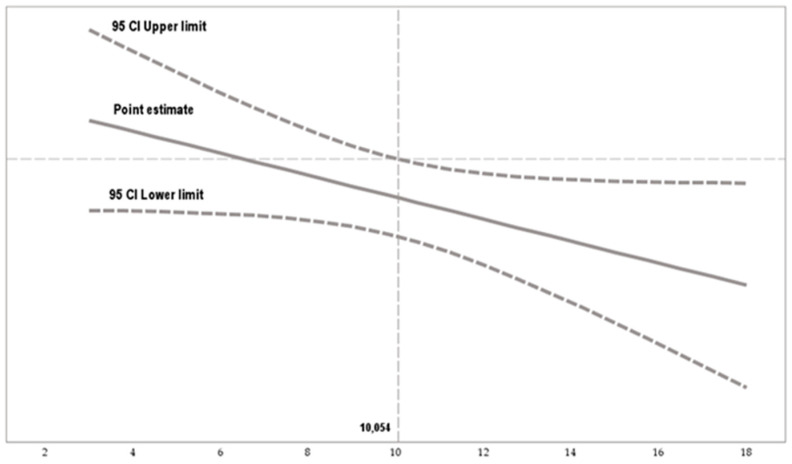
Conditional effect of work autonomy based on the Johnson–Neyman technique. Note. The Johnson–Neyman technique indicates areas of non-significance (interval represented by dashed lines) and areas of significance (interval represented by solid lines).

Figure 3 details the impact of an ethical climate of principles (X) on emotional exhaustion (Y), through the multiple moderating values assumed by work autonomy. Work autonomy changes the orientation of X over Y, starting from a value of 10.054. That is, the influence changes from negative to positive.

**Figure 4:**
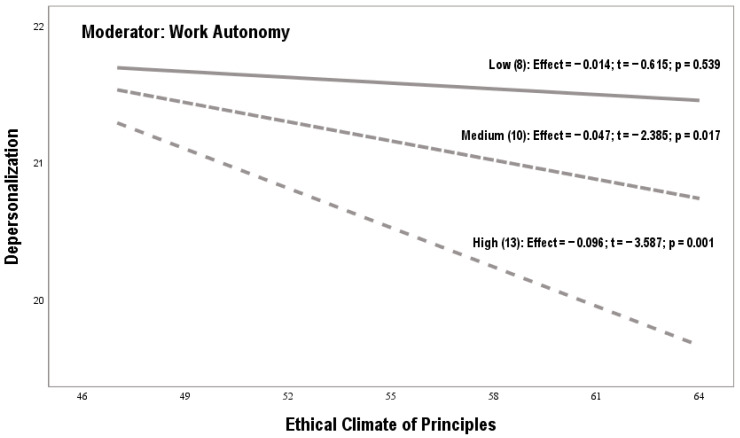
Moderating effect of work autonomy variable (model 2).

Figure 4 shows the moderating effect of work autonomy on the relationship between an ethical climate of principles and depersonalization. The graph shows that low autonomy levels were not statistically significant. In other words, this autonomy range did not influence the impact of an ethical climate of principles (X) on depersonalization (Y). However, a high or medium perception of autonomy changes the meaning of the relationship between X and Y. Therefore when an ethical climate of principles coexists with a medium or high level of autonomy, it has a buffering effect on depersonalization.

**Figure 5:**
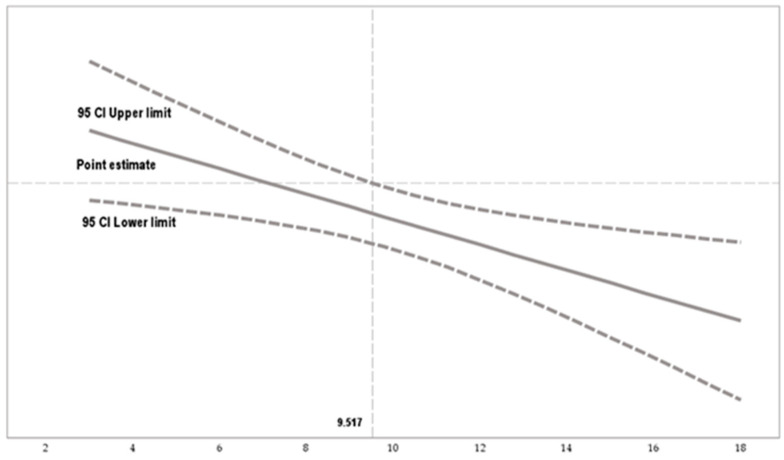
Conditional effect of work autonomy based on the Johnson–Neyman technique.

Figure 5 explains the impact of an ethical climate of principles (X) on depersonalization (Y), through the multiple moderating values assumed by work autonomy. Work autonomy changes the orientation of X over Y, starting from a value of 9.517. That is, the influence changes from adverse to favorable.

## 4. Discussion

This study analyzes the impact of an ethical climate of principles on employees’ emotional exhaustion and depersonalization, considering work autonomy as a relevant moderating factor in this relationship. The findings reveal a positive correlation between an ethical climate of principles and these two subdimensions of the *Maslach Burnout Inventory.* Furthermore, it was observed that the influence of an ethical climate of principles on burnout was conditioned by the level of work autonomy of employees. That is, a higher presence of autonomy was related to lower levels of burnout.

This research makes valuable contributions to the literature for being one of the first articles to detail how an ethical climate based on principles affects employees’ emotional exhaustion and depersonalization, helping fill an important knowledge gap. Although there is information on the effect an ethical climate can have on burnout, for example, Ayub et al. (2022), Rivaz et al. (2020), and Saleh et al. (2022), none of these studies addressed a model focused on the role of an ethical climate of principles as a key component of employees’ emotional well-being. Therefore, the results obtained in this research represent an important step in understanding ethical climates and their effects.

The findings of this research highlight the detrimental effect of an ethical climate of principles on employees’ emotional exhaustion and depersonalization. These results suggest that rules generally enable coherent employee behavior. However, when individual behaviors clash with a highly hierarchical and bureaucratic organizational structure, for instance, the Colombian electric sector with its rigid attitudes towards rules, the independence and dynamic skills needed by professionals tend to be reduced (Tehranineshat et al., 2020). This mechanical, controlled context with little room for proactivity can lead to moral distress and burnout (Lindeberg et al., 2011). In fact, through the development and implementation of its comprehensive anti-corruption program, the Colombian electric sector has defined an ethical climate of principles conditioned by strict policies to counteract bribery, which in turn restricts employee actions (Santiago-Torner, 2023a).

In this sense, Atabay et al. (2015) considered that a rules-based climate may be related to higher levels of moral distress. According to these authors, when employees perceive that their intentions, the product of personal interpretation of what is appropriate behavior, are interrupted by a lack of freedom and feel obliged to follow a path with which they disagree, they repress emotions and consume resources until becoming exhausted. Along with this, Bernuzzi et al. (2022) suggested that moral distress is actively related to emotional exhaustion when it is not refocused positively.

The imposition of a strict work environment, in addition to attacking personal morale, causes professionals to confront this stressor with psychological regulation that tends to the expressive suppression of emotions. This strategy modifies the emotions expressed rather than the internal feelings (Borrelli et al., 2023). Therefore, a maladaptive and contrary effect generates an external and internal discrepancy, which ends in negative emotional experiences. According to the COR theory, a work context that recurrently imposes demands drives subordinates to a progressive energy loss until they become emotionally exhausted, and their emotional well-being deteriorates (Chi & Liang, 2013).

On the other hand, independence to make decisions based on one’s criteria can lead to ignoring formal rules (Vardaman et al., 2014). For example, the Colombian electric sector depends on strong efficiency to solve problems and provide services quickly. Therefore, personal morale may disagree with organizational rules when employees face ethical dilemmas affecting organizational performance, e.g., early delivery of an electrical certification with strong social undertones if such is rapid. A rigid climate stops what personal morale, or codes of conduct consider good intentions, and these types of ethical conflicts can emotionally frustrate employees in addition to requiring more resources. In fact, failure to accept stressors contributes to employees being more vulnerable to emotional exhaustion and depersonalization (Potard & Landais, 2021). Furthermore, the Colombian electric sector faces added complexity as its progress depends on continuous innovation. This static context of a climate with a rigid moral guide, through a set of rules with only one meaning, limits organizational development.

The self-determination theory, previously addressed in this article, suggests that an overly authoritarian and controlling climate limits the basic psychological competencies of autonomy, competence, and relatedness. The work climate is a contextual factor that acts as a resource or demand for the three basic psychological needs. The need for autonomy requires congruence between action and personal values. The need for competence requires a supportive and interactive environment. Finally, the need for relatedness involves a sense of belonging and having significant others nearby. Neglecting the satisfaction of these needs will have a negative impact on the individual psychological development and well-being of the individual. Therefore, a specific context with multiple demands such as that from a rigid and normative ethical climate can become a challenge for the employee that avoids certain positive motivational regulations and makes him/her more susceptible to burnout (Deci et al., 2017; Ryan et al., 2021).

This research has introduced work autonomy as an aspect to explain emotional exhaustion and depersonalization. The results indicate that work autonomy, far from playing a protective role, contributes to increased stress and negative factors linked to the job position. It is important to contextualize these results, considering that the main characteristic of the Colombian electric sector is the high academic training of its personnel. This differential factor, in this specific sector, is likely to turn the work intensity effort into an aspect valued for possible promotions. In this case, work flexibility can become a stressor because tasks control employees. In other words, work autonomy can become a demand when employees lose certain decision freedom (Kubicek et al., 2017).

This result can be explained by “*the autonomy paradox*” proposed by Mazmanian et al. (2013) according to which there is a significant relationship between a greater degree of work autonomy and an increase in the hours dedicated to work, which can lead to a culture of extended work shifts (Shevchuk et al., 2019). Task heterogeneity added to deadlines probably influences the low regulation of the time allocated to their completion. In addition, the Colombian electric sector is obliged to justify all its processes, which entails intense administrative work. The strictness imposed by the sector studied since 2015, with the aim of preventing part of the operation from being diverted into private hands, requires presenting multiple almost immediate reports to demonstrate proper management of the processes.

According to COR theory, work autonomy may compel individuals to alter their interpersonal interactions. Specifically, when autonomy transforms into a demand employees tend to minimize their emotional expenditure by limiting their relationships with others (Hobfoll et al., 2018). Consequently, emotional exhaustion and depersonalization may serve as extreme defense mechanisms against the depletion of emotional resources elucidating the positive correlation between work autonomy and burnout. Conversely, prolonged working hours which arise from the ineffective utilization of autonomy hinder positive interactions with family and friends (Santiago-Torner, 2023a). Furthermore, they diminish the perception of competence as an exhausted individual exhibits reduced efficiency and necessitates additional time to manage the same workload.

Finally, when work autonomy assumes a moderating role, the impact of the ethical climate of principles on emotional exhaustion and depersonalization progressively changes. In other words, a gradual increase in work autonomy reduces the positive impact of an ethical climate of principles on employees’ emotional exhaustion due to the mentioned reasons.

Considering personal morale as a critical organizational aspect prevents employees from intentionally deviating from the norm. Therefore, balancing rules and individual ethical codes will likely influence the employees’ emotional health (Santiago-Torner, 2023b). However, this effect is significant across different perceptions of autonomy (low, medium, and high). First, when an ethical climate of principles coexists with low autonomy, its positive effect on emotional exhaustion and depersonalization does not occur. This scenario is coherent with the model proposed by Karasek (1979), which studies the relationship between the volume of work demands and the degree of autonomy required to handle them. When linked to low autonomy, a rigid and rule-based work climate fosters greater resource consumption, such as extended work shifts. In this context, an ethical climate of principles represents a demand that causes tension and emotional discomfort.

In this regard, the perception of low work autonomy affects at least two of the three fundamental psychological needs outlined by self-determination theory. Specifically low work autonomy restricts the employee’s ability to exert control over their tasks. This situation compels the worker to expend additional emotional resources in an effort to achieve a sense of competence, thereby hindering adequate psychological satisfaction (Ryan et al., 2021). Moreover, the positive correlation between the excessive utilization of resources that the individual is unable to recuperate and the phenomenon of burnout aligns with COR theory (Hobfoll et al., 2018).

Second, when an ethical climate of principles coexists with medium or high autonomy, its positive impact on emotional exhaustion and depersonalization changes from positive to negative. In other words, medium and high work autonomy levels, when combined with an ethical climate of principles, have a buffering effect on emotional exhaustion and depersonalization. Employees under such circumstances can establish a positive relationship between workload and work autonomy, achieving work goals or acquiring new knowledge without it leading to mental health deterioration (Ng & Feldman, 2015). Finally, Zeuge et al. (2023) suggested that education level is a relevant characteristic of the responsible use of autonomy.

### 4.1. Theoretical Implications

Ethical climates serve as fundamental components of organizational functionality as they exert a direct influence on the actions and behaviors of individuals. Indeed, ethical climates can function as valuable instruments for the effective emotional regulation of employees. Nevertheless, to the best of our knowledge, there exists no research that establishes a connection between an ethical climate of principles and burnout taking into account a moderation process, for which our findings are particularly important and contribute significantly to existing knowledge about ethical climates. For instance, Bao et al. (2013) employed the triaxial value model developed by Dolan et al. (2004) to assess ethical climate. Barr (2021), Maffoni et al. (2022), Rivaz et al. (2020), and Tehranineshat et al. (2020) utilized the Hospital Ethical Climate Survey (HECS) scale devised by Olson (1998). Huhtala et al. (2022) evaluated the ethical climate through the corporate ethical virtues questionnaire. Plouffe et al. (2021) implemented the ethical environment questionnaire (McDaniel, 1997). Finally, Saleh et al. (2022) were the pioneering authors to apply one of the scales proposed by Victor and Cullen (1988). However, their research is limited to measuring the ethical climate at the individual level without taking into account other organizational aspects.

Secondly, this study integrates an ethical climate of principles with resource conservation theory and self-determination theory by responding to earlier calls in the literature on the importance of exploring potential mechanisms linking ethical climates with employee health (Delcea et al., 2023). From a resource conservation perspective, our results show that a greater degree of work autonomy changes the relationship between an ethical climate of principles and burnout from positive to negative. Therefore, the integration between an ethical climate of principles and adequate work autonomy provides psychological resources to the employee. Indeed, the role of work autonomy is key to enhancing employee health, e.g., in the workplace by increasing emotional health or reducing stress levels through a greater perception of control over tasks and their possible solutions.

Thirdly, this study offers a robust model that makes a significant contribution to existing knowledge related to human psychology and organizational management. The model of moderation proposed in this article can be replicated in other Latin American countries with similar characteristics to those of Colombia or even transferred to rich countries (Santiago-Torner, 2023b, 2023c).

### 4.2. Practical Implications

This research contributes to the field of organizational health and the prevention of psychosocial risks in the Colombian electrical sector from at least four points of view. First, in a developing country like Colombia and specifically within its electricity sector, an ethical climate of principles can contribute to the development of burnout among employees. This result is particularly valuable since ethical climates have usually been considered as factors that dampen burnout in organizations (Bao et al., 2013; Barr, 2021; Huhtala et al., 2022). This scenario suggests that the set of ethical rules and procedures that define the nature of the Colombian electricity sector rather than becoming a resource, which helps the employee to solve an ethical dilemma, becomes a demand that excessively increases individual stress levels to the point of burnout. Burnout not only affects organizational results through a drastic drop in motivation levels, employee commitment, or productivity but also deteriorates personal health levels through symptoms of chronic fatigue or with recurrent psychosomatic alterations, e.g., the lack of a good health, muscle aches, migraines, gastrointestinal problems, and even dysregulation or loss of the menstrual cycle (Bernuzzi et al., 2022). From the perspective of preventing negative impacts on workers’ health, this result is a wake-up call for the Colombian electricity sector and its managers.

Secondly, several ethics experts agree that burnout comes from moral obligations that are incompatible with the ethical principles of the employee (Hlubocky et al., 2020; Lyon & Galbraith, 2023). Burnout, in addition to producing organizational detachment, induces a loss of empathy, which builds up a cynical and meaningless culture of organizational care. The Colombian electricity sector has the opportunity to balance the ethical discomfort of its employees and reduce their discontent through real policies that respond to real problems. Current trends that prioritize immediacy and outcomes lead to overwork climates that directly impact the physical and mental health of employees. In this sense, autonomy must be regulated through a flexible but limited regulatory framework. That is, the Colombian electricity sector can be key in a systematic change of values that point towards the emotional health of employees.

Thirdly, the Colombian electricity sector is suggested to follow up with internal or external professionals on people diagnosed with burnout. The perception of organizational support often becomes a useful psychological resource for the employee in his recovery period. In addition, the Colombian electricity sector can train its workers in stress management, improving communication skills, or promoting physical and mental self-care.

Fourthly, one of the main objectives of an ethical climate of principles is to encourage members of the organization to report irregularities. However, for this to happen, the Colombian electricity sector needs to increase the perception of organizational support from the employees. Organizational support is an affective variable that bases its effectiveness on the theory of social exchange; that is, employees only respond positively to institutional expectations when they receive favorable treatment from the organization. A strong perception of organizational support can satisfy the emotional needs for self-confidence and belonging that employees possess. It also creates the expectation that greater dedication is recognized and rewarded. Therefore, the Colombian electricity sector should focus its efforts on creating a strong fit between personal and organizational values so that the employee feels valued. This compatibility can improve the mental health of employees (Tartakovsky & Cohen, 2014).

## 5. Limitations and Future Research

This study has several limitations. First, it was cross-sectional and targeted to a specific industrial sector; therefore, the generalization to other sectors may be limited. Second, the findings on the positive relationship between a principled ethical climate, emotional exhaustion, and depersonalization invite to conduct cross-cultural research to establish whether these results are attributable to cultural differences or exclusively to specific organizational practices. Third, the sample is gendered, as 61% of those surveyed were male, and it may be that women’s voices are under-represented in the issues explored. Fourth, although prior sensitization by each participating organization may have helped to mitigate social desirability bias by explaining the importance of answering all questions with complete transparency, the need for social approval is difficult to control when using questionnaires. Finally, only the ethical climate of principles was considered in this study but other ethical climates, e.g., selfish or benevolent, that might be potential predictors of emotional exhaustion and depersonalization need to be taken also into account.

In addition, future research could include other industrial sectors, comprising less masculinized ones, and other moderating effects, such as intrinsic motivation, affective commitment, or psychological empowerment, which could mitigate the impact of emotional exhaustion and depersonalization, as these are in principle individual strengths. Longitudinal studies, through different waves and with intervals equal to or greater than six months, will be very helpful in order to strengthen the conclusions obtained regarding the causality of the relationships among the studied variables. Finally, future research could consider the role of gender and seniority in relation to burnout. The inclusion of gender and seniority as study targets could bring to light certain risks associated with employee physical and emotional health that have not been analyzed in this research.

## 6. Conclusions

The findings of this research, which confirm the three hypotheses that were formulated according to the objective, provide, therefore, empirical evidence for a robust model that makes a significant contribution to existing knowledge related to human psychology and organizational management. The model of moderation proposed in this article can be replicated in other Latin American countries with similar characteristics to those of Colombia or even transferred to rich countries. Furthermore, these findings support the concept of the “*dark side*” of the ethical climate of principles and work autonomy. Disproportionately demanding work environments that address problems through strict rules can lead to inefficient resolutions that frustrate and emotionally exhaust employees. Furthermore, high accessibility to work through the constant use of certain technological advances is likely to cause an imbalance between resources and demands, reversing work autonomy’s positive effect. This prioritization of work to the detriment of life outside work ultimately leads to burnout.

## Figures and Tables

**Table 1:** Descriptive analysis.

Constructs	M	SD	Skewness	Kurtosis
Ethical climate of principles (ECPs) (11)	49.94	2.43	−0.24	1.28
Work autonomy (WA) (3)	1.91	2.54	−0.33	1.27
Emotional exhaustion (EE) (5)	23.11	2.56	−0.40	1.25
Depersonalization (DE) (4)	20.97	3.60	−0.44	1.32

Note: All constructs were evaluated through a 1–6 scale. The range of responses was from “*strongly disagree*” to “*strongly agree*”. The number in brackets indicates the number of items used to measure each construct.

**Table 2:** Correlations between variables and discriminant validity.

Constructs	*N*	ECP	WA	EE	DE
Ethical climate of principles (ECPs)	11	**0.72**			
Work autonomy (WA)	3	0.24 *	**0.89**		
Emotional exhaustion (EE)	5	0.16 *	0.20 *	**0.82**	
Depersonalization (DE)	4	0.23 *	0.14 *	0.59 *	**0.81**

Note: The table shows the Pearson correlations and also includes discriminant validity (bold numbers on the diagonal). (N) Number of items. Significant correlations * (*p* < 0.05). CI (95%) (*n* = 448).

**Table 3:** Model 1, moderation of ethical climate of principles—emotional exhaustion 95% (CI) (R2 = 0.232).

Effect		Route	β	*p*	*t*	SE	LLCI	ULCI
ECP on EE		a1i	0.11	0.01	5.16	0.15	0.15	0.69
WA on EE		a2i	0.57	0.01	5.32	0.25	0.12	0.63
ECP × WA on EE		a3i	−0.02	0.01	−3.62	0.05	−0.05	−0.01
Moderation WA (ECP-EE)	Low (8)		−0.03	0.48	−0.71	0.03	−0.09	0.04
	Med. (10)		−0.06	0.05	−1.94	0.03	−0.12	0.01
	High (13)		−0.11	0.01	−2.65	0.04	−0.19	−0.03

Note. ECPs: ethical climate of principles; EE: emotional exhaustion; WA: work autonomy.

**Table 4:** Model 2, moderation of ethical climate of principles—depersonalization 95% (CI) (R2 = 0.236).

Effect		Route	β	*p*	*t*	SE	LLCI	ULCI
ECP on DE		a1i	0.12	0.01	5.34	0.17	0.23	0.79
WA on DE		a2i	0.69	0.01	4.72	0.35	0.01	1.37
ECP × WA on DE		a3i	−0.02	0.01	−2.75	0.01	−0.03	−0.01
Moderation WA (ECP-DE)	Low (8)		−0.01	0.54	−0.61	0.02	−0.06	0.03
	Med. (10)		−0.05	0.02	−2.38	0.02	−0.08	−0.01
	High (13)		−0.10	0.01	−3.59	0.03	−0.15	−0.04

Note. ECPs: ethical climate of principles; DE: depersonalization; WA: work autonomy.

## Data Availability

The original data presented in the study and the questionnaire used are openly available at The Open Science Framework repository at https://osf.io/w2g5b/?view_only=f8b9995262ed469eab5413f302dd83c4, accessed on 23 January 2025.
